# The Development of Gender Role Attitudes During Adolescence: Effects of Sex, Socioeconomic Background, and Cognitive Abilities

**DOI:** 10.1007/s10964-022-01651-z

**Published:** 2022-07-15

**Authors:** Ricarda Ullrich, Michael Becker, Jan Scharf

**Affiliations:** 1 Department of Educational Governance, DIPF | Leibniz Institute for Research and Information in Education, Rostocker Straße 6, 30323 Frankfurt am Main, Germany; 2 Center for Research on Education and School Development (IFS), Technical University Dortmund, Vogelpothsweg 78, 44227 Dortmund, Germany

**Keywords:** Gender role attitudes, Longitudinal development, Adolescence, Measurement invariance, Growth curve model

## Abstract

How gender role attitudes develop during adolescence, and how biological, social, and cognitive factors predict this development, remains a matter of debate. This study examines the development of gender role attitudes from early adolescence to emerging adulthood and investigates how the developmental trajectory is affected by sex, socioeconomic status, and cognitive abilities (intelligence). Four waves of the large-scale longitudinal German dataset BIJU between 1991 (grade 7; *N* = 3828, *M*_age_ = 13, *SD* = 0.61, 53.1% female, 96.4% German nationality), 1995 (grade 10, *M*_age_ = 17), 1997 (grade 12, *M*_age_ = 19) and 2001/2002 (university/career entry, *M*_age_ = 24) were used. Measurement invariance was examined across waves and gender. Latent growth curve models showed that adolescents developed more egalitarian gender role attitudes. Differences between the sexes decreased over time but remained significant. Socioeconomic status seemed less relevant, while adolescents, especially those with lower intelligence scores, developed more egalitarian gender role attitudes during adolescence. The results showed that teenagers developed more open and egalitarian attitudes during adolescence, and that the development trajectories of female and male adolescents converge.

## Introduction

Gender role attitudes, and the ways in which gender roles are lived out, change not only over time, but also throughout the life course with different ages and contexts (e.g., Dotti Sani & Quaranta, 2017). In particular, adolescence is a period when gender-related constructs, such as gender role attitudes, are especially salient. Teenagers experience biological, cognitive, and social changes during adolescence that can affect their gender role attitudes (Eagly & Wood, 2012). Nevertheless, few studies have addressed how gender role attitudes develop during adolescence. Theoretically as well as empirically, the approaches and results are quite contradictory. Some studies have demonstrated a trend towards more traditional gender role attitudes (e.g., Halimi et al., 2021), while other studies have shown a development towards a more egalitarian direction (e.g., Updegraff et al., 2014). There is also limited research on key predictors of the development of gender role attitudes during adolescence. Using four waves of the BIJU dataset, this study aims to answer the research question of how gender role attitudes develop during adolescence and how this development varies by sex, socioeconomic status, and cognitive abilities.

### The Development of Gender Roles During Adolescence

Gender roles, as a psychological and social construct, comprise both societal expectations and cognitive structures. From a societal perspective, gender roles describe the division of labor and power within a specific cultural and historical context between men and women, with respect to topics such as romantic partnerships, the familial division of labor, and workforce careers. Gender roles are assigned on the basis of sex, traditionally categorized as either male or female. The male role is associated with serving as the family breadwinner, while the female role is associated with social and domestic activities (e.g., Eagly & Wood, 2012). The traditional division of gender roles began to break down in the 20th century, and this process has continued in recent decades. In particular, the female role has undergone substantial change and expanded into areas outside the domestic sphere. As a result, Western-influenced societies have come to exhibit egalitarian gender role attitudes, where both partners share income-earning and domestic and care work on an equal basis (Lomazzi & Seddig, 2020). However, while attitudes are changing, the majority of care work continues to be carried out by women (Zucco & Lott, 2021). Children and adolescents learn through observation that there are societal gender roles, and by internalizing these observations, adolescents develop attitudes towards these gender roles (Eagly & Wood, 2012), which can change over the course of adolescence. However, it remains unclear how gender role attitudes develop during adolescence.

In general, adolescence is an important phase for gender-related changes. Young people discover their individual sexual identity, undergo hormonal and physical changes, and experience their first romantic relationships. Accordingly, the *gender intensification* hypothesis posits that gender role behavior intensifies during adolescence as young people learn to inhabit their later adult roles (including their gender roles) through early experiences with romantic relationships (Hill & Lynch, 1983). This means that traditional gender role attitudes intensify during adolescence. This hypothesis has been confirmed for early adolescent boys, who exhibit an increase in traditional gender role attitudes from grade 7 to grade 8 (Halimi et al., 2021).

In contrast, from a cognitive developmental perspective, it would be argued that the process of discovering one’s own sexuality during adolescence leads young people to question morally built constructs and reconfigure their gender role assumptions (Eccles, 1987). As a result of cognitive maturation, adolescents are able to distinguish between descriptive and prescriptive gender norms and create cognitive representations of newer, more complex social arrangements. Competing concepts can be cognitively integrated to avoid cognitive dissonance, e.g., women can be both loving mothers and have successful careers (Harter, 2003). By investigating gender-based categorization schemes during childhood, research has found that at the end of childhood and the transition to adolescence, previously established categories begin to soften and children no longer rigidly distinguish between male and female characteristics (Trautner et al., 2005). With respect to later development, research has shown that traditional gender role attitudes among Mexican-American boys and girls continuously decline during adolescence (Updegraff et al., 2014). These findings have been confirmed for African-American adolescents (Lam et al., 2017). For Mexican immigrant students, egalitarian gender role attitudes continuously increase across adolescence (Schroeder et al., 2019); this was likewise found for egalitarian attitudes at the end of adolescence, during the transition to adulthood (Fan and Marini 2000). Nevertheless, although traditional gender role attitudes decline initially during adolescence, they can increase towards its end depending on individual and contextual factors (Crouter et al., 2007).

The findings concerning how and to what extent gender role attitudes develop during adolescence are mixed and therefore more research is required. Likewise, it remains unclear which predictors moderate the developmental trajectory. Studies that have examined this question from a developmental perspective have delivered relatively heterogeneous results, partly due to the challenges in the measurement of gender role attitudes. Problematic aspects include modelling one-dimensional scales with egalitarianism at one pole and traditionalism at the other, and capturing temporal dynamics in gender role attitudes, as gender role attitudes change over the individual life course (the focus of this study) while societal gender roles are also changing (Lomazzi, 2017). Consequently, recent studies have sought to test the measurement invariance of the gender role attitudes construct. Internationally comparative studies have shown that complete measurement invariance across countries cannot be assumed. Therefore the use of measurement invariance testing to ensure construct validity before conducting substantive investigations of gender role attitudes is recommend (e.g., Seddig & Lomazzi, 2019). This makes it possible for individual items (e.g., specific questions that might be more or less age-appropriate) to be replaced, or flexible measurement invariance constructs (e.g., partial measurement invariance) to be applied. However, previous studies have not tested the measurement invariance of gender role attitudes in individuals over a longer period of time. Research should investigate whether measurement with a uniform metric is possible over such a long period of individual development, and which restrictions must be placed on measurement invariance assumptions with respect to specific instruments.

### Influences on Gender Role Attitudes

Gender roles are assigned on the basis of supposed biological differences between men and women. These gender roles are taken on by individuals in a society and represent shared normative expectations within a given cultural and historical context (Eagly & Wood, 2012). These gender-based attributions, in turn, are taken up by children as cognitive categories that help structure the social environment (Martin et al., 2002). This study focuses on three key predictive factors for the development of gender role attitudes over time: (1) sex, (2) parents’ socioeconomic status as a key social frame of reference conveying normative attitudes about men and women, and (3) individual cognitive abilities, which influence the categories of cognitive representation that are manifested and expressed in gender role attitudes.

#### Sex differences

Since gender roles are based on the (supposed) biological difference between the sexes, it is important to examine the differences in attitudes towards these roles that emerge between the sexes. Prior research has shown that men and women exhibit different degrees of traditional and egalitarian gender role attitudes (e.g., Bryant, 2003). Traditionally, men and women took on different roles attributed to their biological predispositions. In recent years, as this stereotypical division of roles has broken down, the significance of sex has decreased and the significance of gender as a social construct has increased (Athenstaedt & Alfermann, 2011). This has resulted in a general trend towards more egalitarian attitudes. However, prior research has found evidence for sex differences in such attitudes. Overall, women exhibit more egalitarian attitudes than men (e.g., Bryant, 2003). Internationally comparative research confirms this finding when the social context is considered (Dotti Sani & Quaranta, 2017). Thus, because women are particularly aware of the implications and limitations of the traditional female role, it is especially relevant for them to implement egalitarian structures and endorse egalitarian attitudes, for example, for equal participation in the labor market (Thijs et al., 2019).

Both young women and men develop more egalitarian attitudes during adolescence (e.g., Bryant, 2003). However, there are inconsistent findings with respect to the trajectory of sex differences in gender role attitudes. Some studies have shown that sex differences in gender role attitudes increase during adolescence as female adolescents develop stronger egalitarian attitudes, leading to an increase in sex differences (e.g., Schroeder et al., 2019). However, other studies have shown that male adolescents exhibit a stronger shift towards more egalitarian attitudes during the transition from adolescence to adulthood, leading to a reduction in sex differences (Fan & Marini, 2000). Some studies have found no differences in the two sexes’ trajectories (Updegraff et al., 2014), except regarding the influence of family (Crouter et al., 2007). Overall, the current state of empirical research on the development of sex differences in gender role attitudes can be described as heterogeneous or even contradictory.

#### Social factors

Gender roles represent socially shared assumptions about a certain gendered division of labor and power. Children and adolescents’ first point of reference for the formation and socialization of gender role attitudes is the family. The family is a learning context for gender role behavior, and the family socioeconomic context and parental level of education are predictive of gender role attitudes. Maternal employment and parental occupational prestige influence children’s gender role attitudes, particularly those of girls (McHale et al., 2003). Moreover, a family’s socioeconomic situation is closely linked to children’s aspirations for their future school and career trajectories (Stocké et al., 2011). Girls from socioeconomically privileged households should be particularly likely to develop egalitarian attitudes, as they develop higher aspirations that can only be achieved through egalitarian participation in the labor force (e.g., Mays, 2012).

Families with a high socioeconomic status exhibit more egalitarian gender role attitudes than less socially privileged families. This link can be partially explained by their level of education (e.g., Schroeder et al., 2019), although occupational prestige (particularly of the mother) is also considered relevant (e.g., Lühe et al., 2018). A high level of parental education and maternal employment have a positive effect on teenagers’ egalitarian gender role attitudes. There are differences in effects between male and female adolescents (Fan & Marini 2000). In contrast, research has found that the attitudes of female and male adolescents develop differently over time depending on whether their parents endorse more egalitarian or traditional attitudes. Male adolescents with traditionally oriented parents exhibit almost no changes in attitudes over time. In contrast, a curvilinear trajectory is found for male adolescents with egalitarian-oriented parents. These male adolescents initially develop more egalitarian attitudes, which become more traditional again at the end of adolescence. Female adolescents exhibit a decline in traditional attitudes during adolescence, regardless of their parents’ attitudes. Parents only influence the level of attitudes: female adolescents with more traditional parents also tend to have more traditional attitudes than female adolescents from egalitarian households (Crouter et al., 2007). In summary, the research has shown that higher socioeceonomic status is supportive for egalitarian gender role attitudes among female adolescents, whereas the results for male adolescents are mixed and inconsistent.

#### Cognitive factors

Cognitive factors are the third component that influence gender role attitudes. It can be assumed that higher cognitive abilities are associated with more egalitarian attitudes, as teenagers with higher cognitive abilities are more able to process, reflect, and integrate competing concepts, such as the idea that a woman can be both a good mother and pursue her career ambitions (avoidance of cognitive dissonance: Harter, 2003). This leads to a better understanding of societal structures and potentially critically questioning traditional gender roles (Mays, 2012). Research on cognitive flexibility has demonstrated that children develop more flexible attitudes towards gender stereotypes during the transition to adolescence (Trautner et al., 2005). This is attributed to the fact that children become more cognitively flexible during this period. However, researchers have not been able to empirically investigate any of the cognitive abilities identified as important predictors of change in attitudes during the transition from childhood to adolescence (Trautner et al. 2005).

If one takes cognitive abilities as an increase in education and knowledge, it has been shown that increased education is associated with a stronger preference for egalitarian attitudes, and particularly with a critical view of traditional gender roles (e.g., Bolzendahl & Myers, 2004). Attending college, education in general, and a continuation of education in particular exhibit a significant positive effect on egalitarian gender role attitudes (e.g., Fan & Marini, 2000). However, the association between cognitive abilities and the development of gender role attitudes over time, and the question of whether the effects of cognitive abilities differ between men and women, remains unclear. It might be assumed that women with high cognitive abilities should have a particular interest in the implementation of egalitarian attitudes, which are associated with higher educational aspirations and the pursuit of a career (e.g., Bolzendahl & Myers, 2004). It could also be hypothesized that female adolescents with higher cognitive abilities realize that following egalitarian attitudes towards career orientation and being family orientated may be conflicting goals and conclude that pursuing more traditional attitudes is more advantageous. Moreover, it could be argued that men do not benefit from gender equality or even see it as a threat to their economic position; higher cognitive abilities might not be associated with egalitarian attitudes among men. However, a higher level of education has been shown to be associated with less traditional attitudes among both men and women (Mays, 2012).

## Current Study

Research on developmental trajectories of gender role attitudes and their key influencing factors during adolescence produced ambiguous findings. This study aims to answer the question how gender role attitudes are developing for male and female adolescents and how this trajectory is differing by parents socioeconomic status and cognitive abilities. The first research question is whether gender role attitudes can be measured with a uniform metric over time and sex. The second research question addresses the development of gender role attitudes over time and its associations with the following predictors: sex, socioeconomic status, and cognitive abilities. Based on the aforementioned theoretical considerations and prior empirical findings, it is hypothesized that, in absolute terms, female adolescents should exhibit more egalitarian attitudes than male adolescents at the first measurement point in grade 7 (*M*_age_ = 13). Building upon these assumptions, an exploratory investigation was conducted of whether male and female adolescents exhibit different trajectories and how the difference between the sexes develops over time. Moreover, as the state of research concerning the socioeconomic status and the gender role attitudes is quite heterogeneous (especially for male adolescents), this study examines this correlation exploratively. It is hypothesized that higher individual cognitive abilities are associated with more egalitarian attitudes, as adolescents with higher cognitive abilities should be able to integrate competing concepts more easily. No presumptions are made on how cognitive abilities might predict the developmental trajectory of adolescents’ attitudes over time.

## Methods

### Data

The study on *Educational Careers and Psychosocial Development in Adolescence and Young Adulthood* (BIJU; Baumert et al., 1996) was used for the following analyses. BIJU is a multi-cohort longitudinal study with data collection led by the Max Planck Institute for Human Development in Berlin in cooperation with the Leibniz Institute for Science and Mathematics Education in Kiel. Data collection took place in the German federal states of Mecklenburg-Western Pomerania, North Rhine-Westphalia, and Saxony-Anhalt, and was extended to include schools from Berlin starting in the second wave. Secondary schools from these federal states were sampled, and then two seventh-grade classes were sampled from each of these schools. This resulted in a clustered random sample of *N* = 212 schools with two classes each for the 1991/1992 school year.

The initial seventh-grade sample comprised *N* = 5944 secondary school students. Students from the federal state of Berlin were included in the second wave, increasing the sample size to *N* = 8043. However, the sample size dropped to *N* = 5386 by the fourth wave in grade 10 (1995), as some students left school after obtaining a lower secondary school leaving certificate in grade 9 (*Hauptschulabschluss*) and other students changed schools or were held back a year. Due to the dissolution of existing lower secondary school classes as students entered (university-preparatory) upper secondary school, there was an intentional oversampling of all students in upper secondary schools during the fifth wave (1997, grade 12). This increased the sample size to *N*= 8061. During the sixth wave (2001/2002, university/career entry), data collection took place exclusively by post, reducing the sample size to *N* = 3261 (for more details see Becker et al., 2020).

This study includes the four measurement points at which gender role attitudes were assessed (waves 1, 4, 5 and 6). Students who answered the gender role scale at the first measurement point were used as the sampling basis (*N* = 3837) and were tracked in the following waves (therefore, students from Berlin and the oversampling within the upper secondary schools were excluded through missing by design). Students were excluded if they had either a missing value on the gender and/or weighting variable, resulting in a final sample of *N* = 3828 (Table 1). The sample size dropped to *N* = 1257 by the fourth, *N* = 1167 by the fifth wave and *N* = 732 by the sixth wave (a more detailed attrition analysis is included in the sensitivity analyses). For the selected sample, this led to an overall distribution of participants by gender of 53.8% female and 46.2% male, and the overall weighted distribution of schools was 35.5% academic schools and 64.5% comprehensive schools. From the second wave onwards, 96.4% of the participants were of German nationality, and the students stated at the last wave that 94.9% of their mothers and 94.5% of their fathers were born in Germany.

**Table 1:** Sample sizes from 1991 to 2001/2002

Wave	Year of assessment	Grade	Average age	Sample size
1 (t_1_)	1991	7	13	3828
4 (t_2_)	1995	10	17	1257
5 (t_3_)	1997	12/Vocational training	19	1167
6 (t_4_)	2001/2002	Career/university entry	24	732

Data collection up to students’ graduation (wave 4: grade 10 for vocational education students, wave 5: grade 12 for students enrolled in upper secondary school) took place in the classroom context by trained test administrators. Surveys after students graduated from school (after wave 5 for students out of general upper secondary education, and after wave 6 for all students) were conducted via post; students were asked to provide their addresses for follow-up during the fourth and fifth measurement waves. Written informed consent was obtained from all study participants and their parents, conducted in accordance with the American Psychological Association’s principles for research with human participants. The study was evaluated and approved by the relevant state school boards and the ethics commissions of the participating research institutions (Baumert et al., 1996).

### Measures

#### Gender role attitudes

Gender role attitudes were assessed with attitude-based items measuring gender role orientations (Krampen 1979). The items concerned topics such as romantic partnerships, the family, the workplace, and the rights of men and women (Appendix Table 8). Items addressed an egalitarian division of labor within the family, career ambitions, and normative gender-related attitudes. Responses to all items were recorded on Likert scales ranging from “1 = does not apply at all” to “4 = applies completely”; higher values corresponded to egalitarian attitudes and lower values to non-egalitarian attitudes. The scales exhibited satisfactory to very good reliability scores (wave 1: Cronbach’s *α* = 0.65; wave 4: Cronbach’s *α* = 0.84; wave 5: Cronbach’s α = 0.82; wave 6: Cronbach’s *α* = 0.71). The differences in reliability coefficients are (at least partially) due to the different numbers of items assessed in each wave (range: *N* = 3 to *N* = 7 items) (Hancock & Buehl, 2008).

#### Sex

The data on sex were cleaned to be consistent across waves; boys were coded as 0 and girls as 1.

#### Parental socioeconomic status

Socioeconomic status was assessed with both parental occupational prestige and parental level of education. Four indicators of mothers and fathers’ occupational prestige were employed. Two indicators (one each for mother and father) are based on filled-in information from the first three waves, supplemented by information from the fourth wave. Two additional indicators were used from the fifth wave to control for measurement error. Occupational prestige was coded based on the *International Standard Classification of Occupations* (ISCO-68; International Labour Office, 1968) in waves 1–3 and the ISCO-88 in wave 5 (International Labour Organization, 1990) and converted into the Treiman Prestige Scale (Treiman, 1977). In addition to their parents’ occupations, participants were asked to report the highest level of their parents’ academic and vocational education. Two indicators of parental education were used. First, it was determined whether or not each parent had qualified for higher education (*Abitur*), and second, whether or not each parent had obtained a university degree. Information on both parents was combined: A score of 0 meant that neither parent had an *Abitur*/university degree, while a score of 2 meant that both parents had an *Abitur*/university degree (Becker et al., 2019). For subsequent analyses, these indicators were modelled latently as a time-invariant construct and tested for measurement invariance by sex (Appendix Table 9). Even the model for strict measurement invariance had a very good model fit (RMSEA = 0.04, CFI = 0.98, TLI = 0.98, SRMR = 0.06). Overall, the latent mean value of the socioeconomic status is 5.31 (see also Table 4; by gender males = 5.39 (0.29), females = 5.26 (0.30)).

#### Cognitive abilities

Two different ability tests were employed in the BIJU study to measure the participants’ cognitive abilities. Two subscales on verbal and figural analogies from the *Kognitiver Fähigkeitstest* (KFT; Heller et al., 1985) and two subscales on numeric and spatial reasoning from Amthauer’s (1955) Intelligence Structure Test (IST) were used. The KFT scales exhibited satisfactory reliability (figural: Cronbach’s *α* = 0.93; verbal: Cronbach’s *α* = 0.82). However, the IST scales exhibited only acceptable reliability (spatial: Cronbachs *α* = 0.71/0.70 (Versions A and B); numerical: Cronbach’s *α* = 0.90) (Becker et al. 2020). These four scales were also modeled as a latent intelligence factor for subsequent analysis (Appendix Table 10). A strict level of measurement invariance by sex was confirmed (RMSEA = 0.05, CFI = 1.00, TLI = 1.00, SRMR = 0.04). The cognitive abilities have a latent mean value of 2.44 (see also Table 4; by gender males = 2.39 (0.07), females = 2.52 (0.09)).

### Statistical Analyses

To answer the first research question—whether the gender role attitudes construct can be assessed with the same scale over the entire period from early adolescence to emerging adulthood and whether its developmental trajectory can be modelled with a uniform metric—a latent factor structure of gender role attitudes for each measurement wave was constructed and tested for both longitudinal invariance and multigroup invariance by sex in Mplus 8.4. To compare means on a common metric, and thus to examine questions related to the development of gender role attitudes over time, at least scalar measurement invariance had to be achieved in which both factor loadings and intercepts were constrained to be equal (Meredith, 1993). To achieve longitudinal measurement invariance and measurement invariance by sex, measurement invariance over time and between female and male adolescents was required (Kim & Willson, 2014). For model comparison, the most common absolute measures of model fit (RMSEA, CFI, TLI, and SRMR) were applied, enabling the evaluation of model fit independently of sample size. Model fit was regarded as accepted when the following criteria were met: RMSEA < 0.08, CFI ≥ 0.90, TLI ≥ 0.90, SRMR < 0.08 (Hu & Bentler, 1999; Rutkowski & Svetina, 2014). In addition, changes in the model fit indices across models were evaluated: RMSEA should not increase by a maximum of 0.03 between the configural and metric invariance models, and CFI should not decline by more than 0.02. Between the metric and scalar invariance models, RMSEA should increase by no more than 0.01 and CFI should decrease by no more than 0.01 (Rutkowski & Svetina, 2014).

**Fig. 1:**
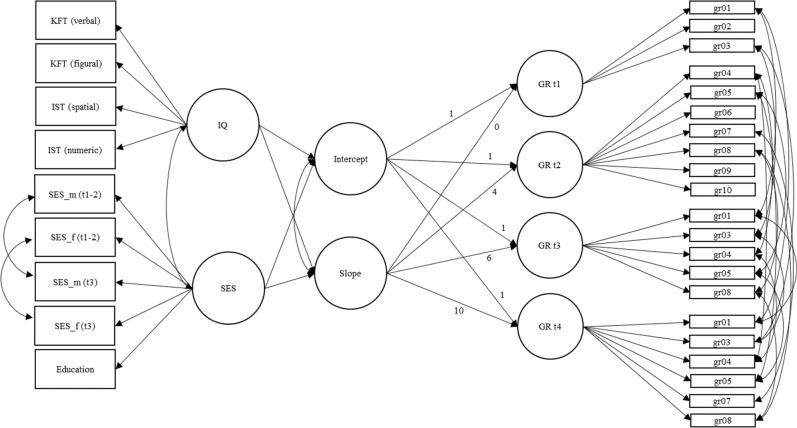
Model of the second-order latent growth curve model and multigroup testing between males and females

To answer the second research question on how gender role attitudes develop during adolescence and which predictors are associated with this trajectory, (latent) means and estimated second-order multigroup latent growth curve models were compared. The mean differences provide an indication of whether any statistically significant changes in means arise between measurement points (wave-specific gender differences). Building upon such changes, growth curve models tested which specific developmental trajectories occur across measurement points and how the included predictors are associated with the *overall* level and trajectory (Duncan et al., 2006; Hancock & Buehl, 2008). Hence, the multigroup second-order model could be used to investigate the development directly in the latent constructs (e.g., Hancock et al., 2001), so the factor loadings and intercepts over time and groups could be constrained while conducting the growth curve model (Fig. 1). The growth curve models were also used to test how socioeconomic status and cognitive abilities predict changes in gender role attitudes and how these two independent variables are associated with initial attitudes in grade 7 (*M*_age_ = 13, Fig. 1). In this context, effects are used in the sense of regression coefficients. Thereby, statements about causality remain open.

Missing values were treated with the full information maximum likelihood (FIML) procedure integrated within Mplus. FIML enables the inclusion of participants with missing values, making full use of the information available in the sample and minimizing the risk of bias in the parameter estimates (Lüdtke et al., 2007). Sampling weights were used to establish a representative proportion of students in academic tracks (Gymnasium) and comprehensive schools. To take the clustered structure of the data into account, the analysis option *type* = *complex* in Mplus was used to estimate standard errors, employing maximum likelihood estimation procedures with robust standard error estimates (mlr).

## Results

### Measurement of Gender Role Attitudes Across Time and Gender

Building upon the first research question examining the presence of a uniform metric for gender role attitudes over time and sex, the factor structure of the egalitarian gender role attitudes scale was tested with a separate confirmatory factor analysis (CFA) for each wave (Table 2). The CFA of the first measurement point was saturated; for the CFAs of the other three waves, wave-specific correlations had to be allowed to adequately represent the items and achieve a satisfactory model fit. For the second measurement point, a correlation between items about whether men or women should enter gender non-conforming professions (gr06 and gr10, see Appendix A7) was allowed. The same applied to measurement time points three and four, where a wave-specific correlation was allowed (in both cases between gr03 and gr05).

**Table 2:** Model fit indices of the gender role attitude scales

Gender role scale	N	RMSEA	CFI	TLI	SRMR
GR t_1_^a^	3828	0.00	1.00	1.00	0.00
GR t_2_	1173	0.10	0.90	0.85	0.05
GR t_2_^b^	1173	0.07	0.95	0.92	0.04
GR t_3_	1106	0.10	0.93	0.86	0.03
GR t_3_^c^	1106	0.05	0.99	0.97	0.02
GR t_4_	729	0.07	0.91	0.86	0.04
GR t_4_^c^	729	0.05	0.96	0.92	0.03

*GR* egalitarian gender role attitudes

^a^Model is saturated

^b^Correlation allowed between items gr06 and gr10

^c^Correlation allowed between items gr03 and gr05

Regarding the test of longitudinal measurement invariance, the configural model exhibited very good model fit (Table 3, Model 1). When constraining the factor loadings (Table 3, Model 2) and intercepts (Table 3, Model 3) to be equal over time, the absolute model fit remained good. The model with the restricted intercepts was maintained and used to test for measurement invariance by sex. As explained in the statistical analyses section, scalar longitudinal measurement invariance was tested and this model was extended step-by-step for measurement invariance by sex (Kim & Willson, 2014).

**Table 3:** Measurement invariance over time and between male and female adolescents

Model	RMSEA	CFI	TLI	SRMR
1 Configural invariance over time	0.02	0.95	0.94	0.05
2 Metric invariance over time	0.02	0.95	0.95	0.06
3 Scalar invariance over time	0.02	0.94	0.93	0.06
4 Scalar invariance over time and configural invariance between groups	0.02	0.90	0.89	0.08
5 Scalar invariance over time and partial configural invariance between groups^a^	0.02	0.92	0.91	0.08
6 Scalar invariance over time and partial configural invariance between groups^b^	0.02	0.93	0.93	0.08
7 Scalar invariance over time and metric invariance between groups	0.02	0.92	0.92	0.09
8 Scalar invariance over time and groups	0.02	0.91	0.90	0.09

*N* = 3828

^a^Correlations allowed between items 4 and 7 for female adolescents and between items 8 and 9 for male adolescents at measurement point 2

^b^Release of intercept gr1 (t1)

The model assuming scalar measurement invariance over time and configural invariance between the sexes (Table 3, Model 4) exhibited unsatisfactory model fit. Based on the modification indices reported in Mplus, one correlation for each sex was allowed at the fourth measurement point (female: gr04 and gr07; males: gr08 and gr09). To further improve the model fit, one constrained intercept for female adolescents from the first measurement point was set free over time (gr01). These model specifications led to a good model fit (scalar measurement invariance over time and partial configural measurement invariance between groups; Table 3, Model 6; Byrne, 2013). In the next step, the factor loadings and intercepts, already fixed over time, were fixed across groups (Table 3, Models 7 and 8). Overall, these models also showed satisfactory absolute fit up to scalar measurement invariance between groups. Thus, with a few limitations, the scale could be confirmed to represent a largely uniform metric over time and across sexes with at least partial measurement invariance. The measurement invariance test was cross-checked with scales containing only items over at least three measurement points. The same pattern emerged (e.g., measurement specific correlations, release of the intercept) and partial scalar measurement invariance over time and between sexes was confirmed (RMSEA = 0.02, CFI = 0.93, TLI = 0.93, SRMR = 0.10).

### Changes in Gender Role Attitudes Across Time

Table 4 shows the bivariate correlations, demonstrating how the constructs of gender role attitudes and the predictor’s sex, socioeconomic status, and cognitive abilities are interrelated. All latent gender role attitudes factors were positively correlated with one another, with particularly strong correlations for the latent factors representing neighboring measurement points. Among the predictors, sex correlated particularly strongly with gender role attitudes; female adolescents displayed more egalitarian attitudes than male adolescents. At the first measurement point only, socioeconomic status correlated positively with egalitarian gender roles, whereas higher cognitive abilities were associated with more egalitarian attitudes at all measurement points. Thus, a more privileged socioeconomic status (only for the first measurement point) and higher cognitive abilities were associated with more egalitarian gender role attitudes. With the exception of the non-significant correlations for socioeconomic status and gender role attitudes at measurement points two, three, and four, all other correlations were in line with the expected pattern.

**Table 4:** Correlations of gender role attitudes and sex, socioeconomic status, and cognitive abilities

Variables	*M* (*SD*)	GR t_1_	GR t_2_	GR t_3_	GR t_4_	Sex	SES
GR t_2_		0.456***					
GR t_3_		0.282***	0.521***				
GR t_4_		0.302***	0.400***	0.483***			
Sex		0.443***	0.473***	0.445***	0.324**		
SES	5.31 (0.30)	0.088***	0.029	0.033	0.015	−0.062**	
IQ	2.44 (0.07)	0.273***	0.230***	0.125**	0.141*	0.067*	0.547***

Sex 0 = male, 1 = female; standardized coefficients

Model fit: RMSEA = 0.02, CFI = 0.95, TLI = 0.95, SRMR = 0.05

*p < 0.05. **p < 0.01. ***p < 0.001

**Fig. 2:**
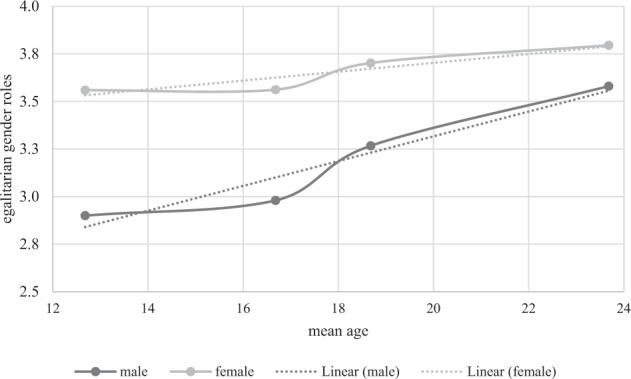
Trajectory of mean values for male and female adolescents during adolescence with trend line

To address the research question of how gender role attitudes develop over time in greater depth, mean comparisons were employed to ascertain the general trajectory of gender role attitudes across waves and gender-specific differences in the trajectory of mean differences across waves. Both genders developed more egalitarian gender role attitudes on average over the measurement points (see Fig. 2, Table 5: male *t*_1_ = 2.944 to *t*_4_ = 3.580; female *t*_1_ = 3.559 to *t*_4_ = 3.795). For both sexes, significant mean changes appeared from the second measurement point onwards, while no significant mean change was evident between the first two measurement points (Table 5, ∆_t-(t-1), males_/∆_t-(t-1), females_). Although no significant change was found between the first two measurement points, the overarching picture indicated that teenagers developed more egalitarian attitudes throughout adolescence.

**Table 5:** Mean differences in gender role attitudes across time and by gender

	Male	Male time Diff(∆)	Female	Female time Diff(∆)	Group Diff(∆)
GR t_1_	2.944 (0.052)		3.559 (0.027)		−0.615***
GR t_2_	2.982 (0.047)	0.038	3.562 (0.040)	0.003	−0.580***
GR t_3_	3.267 (0.043)	0.285***	3.702 (0.017)	0.140***	−0.435***
GR t_4_	3.580 (0.033)	0.313***	3.795 (0.019)	0.093***	−0.215***

*N* = 3828

∆_t-(t-1)_ = Difference score between time *t*-1 and time *t*

Model fit: RMSEA = 0.02, CFI = 0.91, TLI = 0.90, SRMR = 0.09

***p < 0.001

Comparing the means by gender, female adolescents in grade 7 (*M*_age_ = 13) exhibited more egalitarian gender role attitudes than male adolescents (Table 5, ∆_male-female_). This trend continued throughout adolescence, with female adolescents showing more egalitarian gender role attitudes than male adolescents across all measurement points. With respect to the exploratory research question concerning whether male and female adolescents exhibit different developmental trajectories, the mean differences by gender indicate that significant differences remained across all waves but became smaller in magnitude over time (Table 5, ∆_male-female_ = −0.615 to ∆_male-female_ = −0.215).

### SES and Cognitive Abilities as Predictors for Gender Role Attitude Development: Latent Growth Curve Models

To ascertain the extent to which socioeconomic status and cognitive abilities predict the development of gender roles, an overarching developmental curve needs to be aggregated across adolescence. To achieve this, second-order multigroup latent growth curve models were specified with a linear growth curve parameter that considered the different time intervals between measurement waves. Analogously to the mean comparisons, the overarching results across waves (Table 6, Model 1) indicate that both female and male adolescents had a significant positive change coefficient over time. The intercept for female adolescents is higher than for male adolescents, while the slope coefficient is higher for male adolescents than for female adolescents. This confirms the results of the mean comparisons, with female adolescents tending to exhibit more egalitarian initial attitudes than male adolescents, and male adolescents experiencing stronger (positive) changes during adolescence than female adolescents.

**Table 6:** Latent growth curve model for male and female adolescents

		Model 1 *B (SE)*	Model 2 *B (SE)*	Model 3 *B (SE)*	Model 4 *B (SE)*
Model for male adolescents					
	Intercept	2.824 (0.049)***	2.406 (0.191)***	2.436 (0.112)***	2.501 (0.187)***
	Slope	0.071 (0.006)***	0.111 (0.028)***	0.092 (0.017)***	0.101 (0.029)***
SES	Intercept		0.076 (0.034)*		−0.019 (0.047)
	Slope		−0.007 (0.005)		−0.002 (0.006)
IQ	Intercept			0.157 (0.032)***	0.168 (0.046)***
	Slope			−0.010 (0.005)*	−0.009 (0.006)
Variance	Intercept	0.157***	0.154***	0.145***	0.145***
	Slope	0.002*	0.002*	0.002*	0.002*
Model for female adolescents					
	Intercept	3.516 (0.028)***	3.245 (0.136)***	3.093 (0.068)***	3.232 (0.126)***
	Slope	0.027 (0.003)***	0.045 (0.015)**	0.060 (0.008)***	0.044 (0.014)**
SES	Intercept		0.051 (0.024)*		−0.041 (0.028)
	Slope		−0.004 (0.003)		0.005 (0.003)
IQ	Intercept			0.149 (0.021)***	0.174 (0.027)***
	Slope			−0.012 (0.002)***	−0.014 (0.003)***
Variance	Intercept	0.081***	0.078***	0.063**	0.062**
	Slope	0.001	0.001	0.001	0.001

*N* = 3828; male = 1768, female = 2060

*p < 0.05. **p < 0.01. ***p < 0.001

In Model 2, the parental socioeconomic status was added to the model to investigate the extent to which socioeconomic status can predict the development of gender role attitudes. The results show a significant positive effect on gender role attitudes in grade 7 (*M*_age_ = 13) among both male and female adolescents. This indicates that higher parental socioeconomic status is associated with more egalitarian attitudes among teenagers. However, no significant effects of socioeconomic status on the trajectory over time were found in Model 2 for either gender.

As expected, higher cognitive abilities (Model 3) were associated with more egalitarian gender role attitudes in grade 7 (*M*_age_ = 13) for both genders. Regarding the question of how cognitive abilities predict this development, the results show a significant negative effect on the slope. Teenagers with weaker cognitive abilities are particularly likely to develop egalitarian attitudes. In contrast, children with higher cognitive abilities already exhibited more egalitarian attitudes at the beginning of puberty, which did not increase as strongly during adolescence.

When simultaneously considering cognitive abilities and socioeconomic status (Model 4), parental socioeconomic status no longer has a significant effect among male adolescents. However, among female adolescents, comparisons of Model 4 with Model 3 reveal a weak suppression effect, with socioeconomic status exerting a negative effect on initial attitudes in grade 7 (*M*_age_ = 13) and a positive effect on changes during adolescence. Both effects for female adolescents do not reach a sufficient significance level. Controlling for socioeconomic status does not change the pattern of effects of cognitive abilities, but the slope effect for male adolescents becomes insignificant.

### Sensitivity Analyses

The first measurement point was used as a reference and the students were tracked in following waves. Due to the study’s longitudinal design, selective dropout is unavoidable. Dropout by individuals is usually rather systematic, with higher achieving and socially positively selected students showing a higher compliance (e.g., Damian et al., 2015). Therefore, it was tested whether the dropout was systematically related to the constructs being examined. Table 7 documents the sample selectivity of the BIJU study, comparing individuals who participated in the sixth wave with those who no longer participated. As with the aforementioned studies, panel mortality was stronger among students from less socioeconomically privileged households and students with lower cognitive abilities. The selective reduction in the sample is partly due to participants leaving school after the 9th/10th grade, after which these students were only surveyed by post. Moreover, it is important to test whether the sample attrition is related to gender role attitudes. Selective dropout is also discernible here but to a lesser extent. As this attrition has a systematic component related to the constructs of analyses, it is essential to include all individuals in the analyses and not using missing data strategies such as pairwise or listwise deletion, as these rely on more restrictive assumptions for not returning biased estimates (i.e., missing completely at random, which is not the case here; Graham, 2009). FIML was used to retain all students from the first wave in the analyses, which is equivalent to other strategies such as missing data imputation. This minimizes the risk of bias due to selective dropout with respect to the predictors of interest (socioeconomic status and cognitive ability) and maintains maximal test power, as all available information is used.

**Table 7:** Attrition analysis for students who participated or did not participate in the last wave, comparing predictors of cognitive abilities, socioeconomic status, and gender role attitudes

	Overall			Participation W6 (t_4_)			No participation W6 (t_4_)						
Construct	*N*	*M*	*SD*	*N*	*M*	*SD*	*N*	*M*	*SD*	*F*	*df*	*p*	*d*
IST numeric	3071	9,49	5,39	682	11,89	5,13	2389	8,81	5,27	0,26	3069	<0.001	0,57
IST spatial	3097	6,97	3,89	687	8,47	2,92	2410	6,54	3,00	2,32	3095	<0.001	0,50
KFT figural	3409	0,59	1,70	686	1,39	1,49	2723	0,39	1,70	32,95	3407	<0.001	0,59
KFT verbal	3481	9,69	4,76	662	12,16	4,67	2819	9,11	4,60	0,95	3479	<0.001	0,64
SES mother (wave 1–3, 4)	2792	44,87	12,69	608	48,25	12,18	2184	43,93	12,68	0,07	2790	<0.001	0,34
SES father (wave 1–3, 4)	2821	44,56	13,01	655	47,51	13,87	2166	43,67	12,60	28,50	2891	<0.001	0,30
Parents‘ education	3636	0,78	0,86	730	1,21	0,84	2906	0,67	0,84	0,15	3634	<0.001	0,63
GR t_1_	3828	3,29	0,70	732	3,42	0,63	3096	3,26	0,71	21,56	3826	<0.001	0,22
GR t_2_	1173	3,24	0,58	583	3,31	0,57	590	3,17	0,59	1,18	1171	<0.001	0,24
GR t_3_	1106	3,57	0,49	659	3,62	0,43	447	3,48	0,56	39,73	1104	<0.001	0,30

To check for robustness, the results were further replicated with an even more inclusive data strategy using the complete sample. This included students from the federal state of Berlin who entered the study from the second wave onwards, students who joined the study through restructured class compositions (especially in the fourth wave/10th grade when many students entered the original classes), and students from an intentional oversample assessing all students within upper secondary schools during the fifth wave (grade 12, Mage = 19). This increased the test power of the sample to a sample size of *N* = 11,713. This sample is less representative, mainly due to grade 12 oversampling. Using this sample, analyses showed a similar pattern (Appendix Fig. 3), except that the socioeconomic status correlated significantly with gender role attitudes at all measurement points. This is most likely due to the higher test power in the extended sample.

A second analysis was conducted with participants who rated the gender role attitudes scale at all measurement points (i.e., relying on a casewise deletion strategy; *N* = 561). The overall developmental pattern of gender role attitudes remained the same for male and female adolescents (Appendix Fig. 4). No significant effects were found regarding socioeconomic status and cognitive abilities. However, it is unclear whether this is due to the more restrictively selected sample, the reduced test power, or even biased due to the assumptions this sample selection makes (missing completely at random which does not apply here; see dropout analyses, Table 7).

Since gender roles are a changing construct, it is sometimes necessary to exchange indicators in longitudinal studies to ensure that they continue to represent sufficient variance (see also gender role scales of the European Values Study, EVS, 2021). To determine whether measurement invariance is just an artefact, analyses were conducted in which the items were linked across at least three measurement points. Scalar invariance over time and groups was achieved without any partial adjustments (RMSEA = 0.02, CFI = 0.91, TLI = 0.91, SRMR = 0.10). The following developmental analyses confirmed the pattern of the analyses with the complete scales.

## Discussion

Gender role attitudes develop during adolescence. However, previous studies have shown inconsistent findings, and no study has yet examined the full period from early adolescence through emerging adulthood. This study investigated the development of gender role attitudes across the entirety of adolescence against the backdrop of existing societal gender differences. Adolescence is particularly relevant, as gender-related constructs are especially salient during this time. The extent to which greater endorsement of egalitarian gender role attitudes is associated with sex, socioeconomic status, and individual cognitive abilities was analyzed with a series of structural equation models to further evaluate the measurement models and build upon and expand prior studies on the appropriate modelling of gender role attitudes (e.g., Lomazzi & Seddig, 2020).

Overall, the results indicate that both male and female adolescents develop more egalitarian gender role attitudes during adolescence. This finding can be linked to the assumption that young people increasingly question morally built constructs, leading to the endorsement of more egalitarian gender roles (Eccles, 1987). Moreover, it confirms prior research that has shown that egalitarian attitudes increase during adolescence (e.g., Schroeder et al., 2019). Consequently, the finding that boys experience an increase in traditional gender role attitudes during early adolescence was not replicated (Halimi et al., 2021). Likewise, the opposing *gender intensification hypothesis*—that gender role behavior increases during adolescence and sex differences increase (Hill & Lynch, 1983)—was not supported. Although teenagers discover their gender identity during this time, the results show that attitudes towards gender roles nevertheless soften, and explicit role attributions are less supported. Moreover, contrary to the *gender intensification hypothesis*, male adolescents experience greater change towards egalitarian direction than female adolescents. This reduces gender differences, although they remain at a significant level. Thus, endorsing egalitarian attitudes seems to be particularly important for women. To participate equally in the labor market, it is particularly relevant for women to pursue egalitarian attitudes, as they are traditionally assigned the domestic role. When female adolescents begin to consider their future plans (which first include decisions on careers after school), egalitarian attitudes are particularly relevant. While egalitarian attitudes are especially important for women, male adolescents develop more strongly towards an open and egalitarian direction. Thus, the finding that sex differences in gender role attitudes decline during adolescence (Fan & Marini, 2000) was replicated.

Cognitive abilities were found to have significant positive effects on egalitarian gender role attitudes. This could be an indication that young people with higher cognitive abilities are better able to process seemingly competing concepts and critically question social structures, including critically reflecting on traditional gender role attitudes (Harter, 2003). No gender differences were found with respect to this relationship; higher cognitive abilities promote egalitarian attitudes among both male and female adolescents. However, a negative slope effect was found, indicating that young people with weaker cognitive abilities are particularly likely to develop more egalitarian attitudes over time.

More ambiguous results were found for the effects of family background. Socioeconomic status correlated positively with gender role attitudes only at the first measurement point. Moreover, no significant slope effect was found, and when controlling for cognitive abilities, the significant intercept effect of parental socioeconomic status became insignificant. Despite the presence of a link between family socioeconomic background and gender role attitudes, this factor is not predictive for changes during adolescence. This may be because young people become more independent of their family during adolescence, distancing themselves from certain attitudes imparted within the family. A more important contextual factor than the family could be peer groups and school classes, which form a primary point of reference for teenagers (Halimi et al., 2021). In particular, gender-related attitudes of peer groups could be relevant in the formation of gender identity and gender role attitudes. Also, by the end of the study period the adolescents had already entered adulthood and their own socioeconomic status may have become more relevant than their parents’ socioeconomic status. The study demonstrates that gender role attitudes experience changes during adolescence, confirming prior results that have observed an increase in egalitarian attitudes over the course of adolescence (Eccles, 1987). That these trajectories converge during adolescence seems to be a central and overarching aspect of development.

### Limitations

The longitudinal dataset employed in this study provided an overview of the development of gender role attitudes across the entire period of adolescence. It captured long-term developmental trajectories from early adolescence to emerging adulthood while using an extensive sample (*N* = 3828). A partially uniform metric was applied over time and across groups to measure the development of gender role attitudes over time. Despite these advantages, the study also exhibited several limitations with relevant implications for future research.

Some challenges arose when attempting to model the gender role attitudes construct in this study. Previous research (e.g., Knight & Brinton, 2017) has shown that gender roles cannot necessarily be mapped on a one-dimensional scale with egalitarianism at one end and traditionalism at the other. The item statements used to assess gender role attitudes (Appendix Table 8) encompass both descriptive statements on how men and women actually relate to one another and prescriptive statements about how they *ought* to relate to one another (Krampen, 1979). In addition, gender role attitudes can be divided into different facets. They encompass models for dividing domestic and paid labor among couples, while also including normative and legal aspects of gender equality and women’s greater presence in public life (Constantin & Voicu, 2015). This multidimensional perspective on gender role attitudes cannot always be converted into a one-dimensional scale with egalitarianism at one end and traditionalism at the other. These various facets of gender role attitudes are also contained within the construct used here. A scale measuring egalitarian gender role attitudes was employed for two key reasons: first, due to the increasing endorsement of egalitarian attitudes in Western societies (Lomazzi & Seddig, 2020), and second, because the egalitarian attitudes scale included sufficient linkages between items across measurement waves to examine developmental trajectories. Nevertheless, the use of the scale may have had an effect on the results, as acquiescence led adolescents to agree more with egalitarian statements, leading to the rejection of the *gender intensification hypothesis*.

Following recent recommendations, the egalitarian gender role attitudes scale was embedded in structural equation models and tested for measurement variance over time and across genders (e.g., Lomazzi & Seddig, 2020). This study was able to partially confirm the scale’s measurement invariance in both ways. However, due to the aforementioned complexity, recourse to partial measurement invariance was unavoidable in some cases (Byrne, 2013). Moreover, the indicators shifted across measurement points, as the number of items measuring egalitarian attitudes was lower in the beginning and increased over time. This meant that only two anchor items were available for the first measurement point (Hancock & Buehl, 2008). However, the exchange of items over time is not necessarily avoidable in a longitudinal perspective with a changing social construct. Agreement with items, such as that women should have the same rights as men, reaches a ceiling by no longer reflecting variance after a certain point in time (see also gender role scales of the European Values Study, EVS, 2021). However, the sensitivity analysis concerning the shorter scale showed that measurement invariance and the developmental pattern were confirmed.

It was not possible to model a quadratic slope in the latent growth curve models due to convergence problems. This might have been due to the relatively low variance of the slope parameters. This issue could not be solved with the presented models because the most common solutions (e.g., fixing the residuals of the same indicators over time) did not achieve satisfactory model fit. This may be a further indication that modelling gender role attitudes remains a key issue requiring more extensive and in-depth research.

A typical issue of longitudinal analyses, which also affected this study, is panel attrition. People with lower cognitive abilities and socioeconomic status are more likely to drop out of the study. This is particularly relevant in this context, as these are key predictors of the developmental trajectory. Therefore, it is important to use missing data strategies such as FIML, as this strategy enables the retention of all students. Thereby, all existing information is used, maintaining the test power and minimizing the risk of selective dropout (Graham, 2009). Nevertheless, the generalizability should be interpreted with caution and the effects may be underestimated. Further replications with other data sets are needed to test the robustness of the findings presented here.

It was also not possible to control for relevant predictors. No data was available from the parents themselves, so there was no information on the gender role attitudes of the parents. Moreover, it was not possible to look at time-variant confounders like biological changes (e.g., hormonal changes) or the time point when the participants had their first romantic and sexual experiences. Likewise, time-invariant confounders, such as genes or personality traits, could not be considered. Future research should consider whether these could be relevant and specific predictors for the development of gender role attitudes.

Lastly, the results need to be discussed from a historical perspective as the data basis refers to the 1990s and 2000s. In the last decade, societal discourses have increasingly engaged topics such as #metoo, nonbinary gender identities, and new ways to understand masculinity (Walter, 2018). This discourse is currently being led by a (publicly very present) section of adolescents and young adults with an intensity that was not as characteristic and polarizing for the same age group in the 1990s. Nevertheless, the adolescents in this study developed more egalitarian attitudes, and this can also be assumed for today’s teenagers based on current debates. The results highlight the changes in attitudes towards gender roles that take place during adolescence, and the importance of this perspective when studying gender inequalities. Although the data refers to the 1990s, there are few research approaches and datasets with a developmental perspective on the whole of adolescence.

### Implications

This study has key implications for future research. Methodologically, increased latent modelling of gender role attitudes combined with extensive measurement invariance testing is required to adequately deal with shifting indicators. First, social change needs to be reflected in attitudes towards gender roles so that adequate variance can be modelled. Second, young people’s attitudes to gender roles change as they move through adolescence. While young people in the seventh grade may only be observers of their parents, they will have already made occupational decisions by the end of the study period that may go hand in hand with their gender roles. Moreover, future research should compare the development of traditional and egalitarian gender role attitudes to separate descriptive and prescriptive parts of the items. Intensive content and methodological research on the development of attitudes towards gender roles in adolescence is required, as the results show that adolescents develop in an egalitarian direction, while gender differences continue to emerge in occupational decisions. This may clarify how gender differences manifest early on.

## Conclusion

Attitudes towards gender roles change during adolescence, yet the state of research is limited and inconsistent. This study investigated how gender role attitudes develop during adolescence and whether the trajectories differ by gender, socioeconomic status of the parents and cognitive abilities. The results highlight that both male and female adolescents developed egalitarian gender roles during adolescence and that their trajectories were converging by the end of the measurement period, leading to the rejection of the *gender intensification hypothesis* (Hill & Lynch, 1983). The assumption was supported that adolescents increasingly question moral constructs and, accordingly, develop in a more egalitarian direction (Eccles, 1987). In particular, cognitive abilities play an important role in egalitarian gender role attitudes. Studying the development of gender role attitudes is important in understanding gender inequalities, as gender-related constructs are particularly salient during adolescence, when teenagers discover their own gender identity and lay important foundations for their occupational and family-related futures. Since the data set refers to the 1990s, it would be useful to compare how attitudes towards gender roles develop during adolescence in the context of current social debates and changes to separate cohort effects from individual development processes. This study can serve as a central point of reference in this endeavor.

## Figures and Tables

**Fig. 3:**
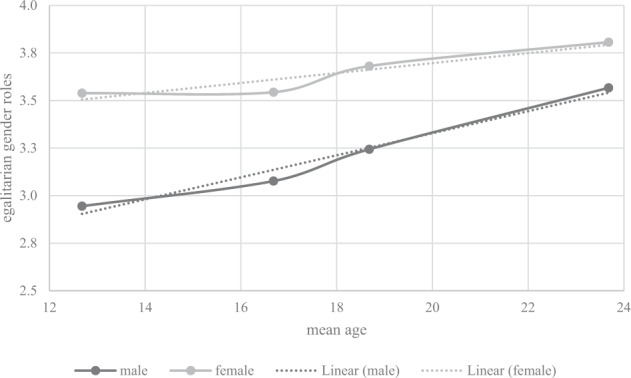
Trajectory of mean values for male and female adolescents during adolescence with trend line. *N* = 11713

**Fig. 4:**
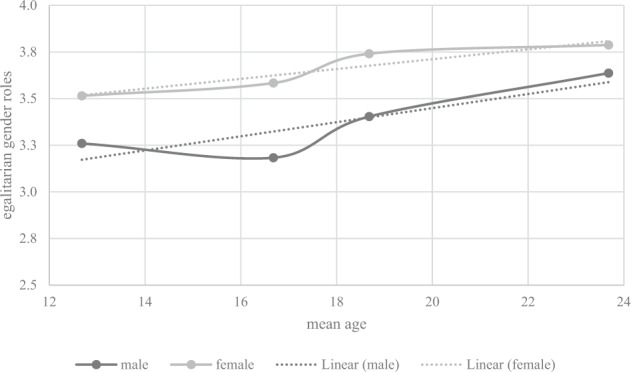
Trajectory of mean values for male and female adolescents during adolescence with trend line. *N* = 561

## Data Availability

We used Mplus 8.4 to model the latent structure of our interested factors. Furthermore, we tested for longitudinal measurement invariance and measurement invariance by sex and we modeled our final multigroup second order latent growth curve model with Mplus 8.4. The related code is available by contacting the corresponding author: ullrich@dipf.de.

## Appendix

Tables 8–10

**Table 8:** Gender role attitudes items across waves

Items	Description: 4-point Likert scale from 1 (does not apply at all) to 4 (applies completely)	Wave 1 (t_1_)	Wave 4 (t_2_)	Wave 5 (t_3_)	Wave 6 (t_4_)
gr01	Im Durchschnitt sind Mädchen so klug wie Jungen. *On average, girls are as smart as boys*.	x		x	x
gr02	Wenn Mann und Frau beide berufstätig sind, sollte der Mann einen Teil der Hausarbeit übernehmen wie etwa Geschirrspülen und Waschen. *If a man and woman are both employed, the man should do some of the housework, such as washing the dishes and laundry*.	x			
gr03	Mädchen sollten dieselben Freiheiten haben wie Jungen. *Girls should have the same freedoms as boys*.	x		x	x
gr04	Wenn Frau und Mann beide berufstätig sind, sollten sie auch die Hausarbeit und Kindererziehung zu gleichen Teilen übernehmen. *If women and men are both employed, they should also share the housework and child rearing equally*.		x	x	x
gr05	Männer sollten nicht nur auf beruflichen Erfolg aus sein, sondern auch ein gutes Familienleben als Erfolg ansehen. *Men should not only seek out professional success, but should also consider a good family life as success*.		x	x	x
gr06	Frauen sollten auch traditionell männliche Berufe wie Ingenieur oder Schlosser ergreifen. *Women should also take up traditionally male occupations such as engineer or locksmith*.		x		
gr07	Frauen sollte es genauso wichtig sein wie Männern, im Beruf Karriere zu machen. *It should be just as important for women to have a successful career as it is for men*.		x		x
gr08	Ein Mann sollte kein schlechtes Gewissen haben, wenn seine Frau mehr verdient als er. *A man should not feel guilty if his wife earns more than he does*.		x	x	x
gr09	Es ist egal, ob Vater oder Mutter die Betreuung der Kinder übernimmt und dafür im Beruf zurücksteckt. *It doesn’t matter whether the father or mother serves as primary caregiver for the children and cuts back on his or her career in return*.		x		
gr10	Männer sollten ruhig auch Berufe ergreifen, die traditionell Frauen vorbehalten sind (z.B. Kindergärtner, Krankenpfleger). *Men should feel free to enter occupations traditionally reserved for women (e.g., nursery school teacher, nurse)*.		x

**Table 9:** Measurement invariance of socioeconomic status by gender

Model	RMSEA	CFI	TLI	SRMR
Configural invariance	0.06	0.99	0.95	0.03
Metric invariance	0.04	0.98	0.97	0.06
Scalar invariance	0.04	0.98	0.98	0.05
Residual invariance	0.04	0.98	0.98	0.06

*N* = 3788; male = 1743, female = 2045

**Table 10:** Measurement invariance of cognitive abilities by gender

Model	RMSEA	CFI	TLI	SRMR
Configural invariance	0.03	1.00	1.00	0.01
Metric invariance	0.02	1.00	1.00	0.02
Scalar invariance	0.06	1.00	1.00	0.04
Residual invariance	0.05	1.00	1.00	0.04

*N* = 3778; male = 1748, female = 2030

Figs. 3–4

## References

[CR1] Amthauer, R. (1955). *I-S-T: Intelligenz-Struktur-Test: Handanweisung für die Durchführung und Auswertung* (2nd ed.). Hogrefe.

[CR2] Athenstaedt, U., & Alfermann, D. (2011). *Geschlechtsrrollen und ihre Folgen: Eine sozialpsychologische Betrachtung*. Kohlhammer.

[CR3] Baumert, J., Roeder, P. M., Gruehn, S., Heyn, S., Köller, O., Rimmele, R., Schnabel, K., & Seipp, B. (1996). Bildungsverläufe und psychosoziale Entwicklung im Jugendalter (BIJU). In K.-P. Treumann, G. Neubauer, R. Möller, & J. Abel (Eds.), *Methoden und Anwendungen empirischer pädagogischer Forschung* (pp. 170–180). Waxmann.

[CR4] Becker M, Baumert J, Tetzner J, Maaz K, Köller O (2019). Childhood intelligence, family background, and gender as drivers of socioeconomic success: The mediating role of education. Developmental Psychology.

[CR5] Becker M, Tetzner J, Baumert J (2020). Schulformen und sozioökonomischer Erfolg im jungen Erwachsenenalter: Werden unterschiedliche Ausbildungswege auf dem Arbeitsmarkt gleich honoriert. Zeitschrift Für Erziehungswissenschaft.

[CR6] Bolzendahl CI, Myers DJ (2004). Feminist attitudes and support for gender equality: Opinion change in women and men, 1974-1998. Social Forces.

[CR7] Bryant AN (2003). Changes in attitudes towards woman’s roles: Predicting gender-role traditionalism among college students. Sex Roles.

[CR8] Byrne, B. M. (2013). *Structural equation modeling with Mplus: Basic concepts, applications, and programming*. Routledge.

[CR9] Constantin A, Voicu M (2015). Attitudes towards gender roles in cross-cultural surveys: Content validity and cross-cultural measurement invariance. Social Indicator Research.

[CR10] Crouter AC, Whiteman SD, McHale SM, Osgood DW (2007). Development of gender attitude traditionality across middle childhood and adolescence. Child Development.

[CR11] Damian RI, Su R, Shanahan M, Trautwein U, Roberts BW (2015). Can personality traits and intelligence compensate for background disadvantage? Predicting status attainment in adulthood. Journal of Personality and Social Psychology.

[CR12] Dotti Sani GM, Quaranta M (2017). The best is yet to come? Attitudes toward gender roles among adolescents in 36 countries. Sex Roles.

[CR13] Duncan, T. E., Duncan, S. C., & Strycker, L. A. (2006). *An introduction to latent variable growth curve modeling: Concepts, issues and applications*. Lawrence Erlbaum Associates.

[CR14] Eagly, A. H., & Wood, W. (2012). Social role theory. In Van Lange, Paul A. M., A. W. Kruglanski, & E. T. Higgings (Eds.), *Handbook of theories of social psychology: Volume 2* (pp. 458–476). Sage.

[CR15] Eccles, J. (1987). Adolescence: Gateway to gender-role transcendence. In D. B. Carter (Ed.), *Current conceptions of sex roles and sex typing: Theory and Research* (pp. 225–242). Praeger.

[CR16] EVS. (2021). *EVS Trend File 1981-2017*. 10.4232/1.13736

[CR17] Fan P‑L, Marini MM (2000). Influences on gender-role attitudes during the transition to adulthood. Social Science Research.

[CR18] Graham JW (2009). Missing data analysis: Making it work in the real world. Annual Review of Psychology.

[CR19] Halimi M, Davis SN, Consuegra E (2021). The power of peers? early adolescent gender typicality, peer relations, and gender role attitudes in Belgium. Gender Issues.

[CR20] Hancock GR, Buehl MM (2008). Second-order latent growth models with shifting indicators. Journal of Modern Applied Statistical Methods.

[CR21] Hancock GR, Kuo W‑L, Lawrence F (2001). An illustration of second-order latent growth models. Structural Equation Modeling: A Multidisciplinary Journal.

[CR22] Harter, S. (2003). The development of self-representations during childhood and adolescence. In M. R. Leary & J. P. Tangney (Eds.), *Handbook of self and identity* (610-642). Guilford Press.

[CR23] Heller, K. A., Schoen-Gaedike, A.‑K., & Weinlaeder, H. (1985). *Kognitiver Fähigkeitstest: KFT 4-13+* (2nd ed.). Beltz.

[CR24] Hill, J. P., & Lynch, M. E. (1983). The intensification of gender-related role expectations during early adolescence. In J. Brooks-Gunn & A. C. Petersen (Eds.), *Girls at puberty: Biological and psychosocial perspectives* (pp. 201–228). Springer.

[CR25] Hu L, Bentler PM (1999). Cutoff criteria for fit indexes in covariance structure analysis: Conventional criteria versus new alternatives. Structural Equation Modeling: A Multidisciplinary Journal.

[CR26] International Labour Office. (1968). *International Standard Classification of Occupations: Revised edition*. International Labour Office.

[CR27] International Labour Organization. (1990). *International standard classification of occupations: ISCO-88*. International Labour Organization.

[CR28] Kim ES, Willson VL (2014). Testing measurement invariance across groups in longitudinal data: multigroup second-order latent growth model. Structural Equation Modeling: A Multidisciplinary Journal.

[CR29] Knight CR, Brinton MC (2017). One egalitarianism or several? Two decades of gender-role attitude change in Europe. American Journal of Sociology.

[CR30] Krampen G (1979). Eine Skala zur Messung der normativen Geschlechtsrollen-Orientierung (GRO-Skala). Zeitschrift Für Soziologie.

[CR31] Lam CB, Stanik C, McHale SM (2017). The development and correlates of gender role attitudes in African American youth. The British Journal of Developmental Psychology.

[CR32] Lomazzi V (2017). Testing the goodness of the EVS gender role attitudes scale. Bulletin of Sociological Methodology/Bulletin De Méthodologie Sociologique.

[CR33] Lomazzi V, Seddig D (2020). Gender role attitudes in the international social survey programme: cross-national comparability and relationships to cultural values. Cross-Cultural Research.

[CR34] Lüdtke O, Robitzsch A, Trautwein U, Köller O (2007). Umgang mit fehlenden Werten in der psychologischen Forschung. Psychologische Rundschau.

[CR35] Lühe J, Becker M, Maaz K (2018). Elterliche Geschlechterrollenvorstellungen, familiärer Hintergrund und Schulleistungen. Zeitschrift Für Pädagogische Psychologie.

[CR36] Martin CL, Ruble DN, Szkrybalo J (2002). Cognitive theories of early gender development. Psychological Bulletin.

[CR37] Mays A (2012). Determinanten traditionell-sexistischer Einstellungen in Deutschland – eine Analyse mit Allbus-Daten. Kölner Zeitschrift Für Soziologie Und Sozialpsychologie.

[CR38] McHale SM, Crouter AC, Whiteman SD (2003). The family context of gender development in childhood and adolescence. Social Development.

[CR39] Meredith W (1993). Measurement invariance, factor analysis and factorial invariance. Psychometrika.

[CR40] Rutkowski L, Svetina D (2014). Assessing the hypothesis of measurement invariance in the context of large-scale international surveys. Educational and Psychological Measurement.

[CR41] Schroeder KM, Bámaca-Colbert MY, Robins RW (2019). Becoming more egalitarian: a longitudinal examination of Mexican-origin adolescents’ gender role attitudes. Developmental Psychology.

[CR42] Seddig D, Lomazzi V (2019). Using cultural and structural indicators to explain measurement noninvariance in gender role attitudes with multilevel structural equation modeling. Social Science Research.

[CR43] Stocké V, Blossfeld H‑P, Hoenig K, Sixt M (2011). Social inequality and educational decisions in the life course. Zeitschrift Für Erziehungswissenschaft.

[CR44] Thijs P, Te Grotenhuis M, Scheepers P, van den Brink M (2019). The rise in support for gender egalitarianism in the Netherlands, 1979-2006: The roles of educational expansion, secularization, and female labor force participation. Sex Roles.

[CR45] Trautner HM, Ruble DN, Cyphers L, Kirsten B, Behrendt R, Hartmann P (2005). Rigidity and flexibility of gender stereotypes in childhood: Developmental or differential. Infant and Child Development.

[CR46] Treiman, D. J. (1977). *Occupational prestige in comparative perspective*. Academic Press.

[CR47] Updegraff KA, McHale SM, Zeiders KH, Umaña-Taylor AJ, Perez-Brena NJ, Wheeler LA, Rodríguez De Jesús SA (2014). Mexican-American adolescents’ gender role attitude development: The role of adolescents’ gender and nativity and parents’ gender role attitudes. Journal of Youth and Adolescence.

[CR48] Walter JG (2018). The adequacy of measures of gender roles attitudes: A review of current measures in omnibus surveys. Quality & Quantity.

[CR49] Zucco A, Lott Y (2021). Stand der Gleichstellung: Ein Jahr Corona (WSI Report No. 64).

